# Differential Modulation of the Excitatory and Inhibitory Synaptic Circuits of Retinal Ganglion Cells via Asiatic Acid in a Chronic Glaucoma Rat Model

**DOI:** 10.3390/jcm12031056

**Published:** 2023-01-29

**Authors:** Yinglei Zhang, Chunyan Hu, Cong Niu, Jiaxu Hong, Xujiao Zhou

**Affiliations:** 1 Eye Institute, Eye & ENT Hospital, Fudan University, Shanghai 200032, China; 2 Shanghai Key Laboratory of Visual Impairment and Restoration, Shanghai 200032, China; 3 NHC Key Laboratory of Myopia, Eye & ENT Hospital, Fudan University, Shanghai 200032, China; 4 Key Laboratory of Myopia, Chinese Academy of Medical Sciences, Shanghai 200032, China; 5 Department of Pathology, Eye & ENT Hospital, Fudan University, Shanghai 200032, China; 6 Department of Respiratory Medicine, Huadong Hospital, Fudan University, Shanghai 200040, China

**Keywords:** asiatic acid, excitatory synaptic circuits, inhibitory synaptic circuits, retinal ganglion cells, glaucoma

## Abstract

Purpose: To investigate whether asiatic acid (AA) can improve the quantity and function of retinal ganglion cells (RGCs), as well as how AA regulates synaptic pathways in rat models with chronic glaucoma. Methods: In our study, a rat model of chronic glaucoma was prepared via the electrocoagulation of the episcleral veins. The numbers of surviving RGCs were counted via retrograde Fluorogold labeling, and a whole-cell patch clamp was used to clamp RGCs in normal retinal sections and in retinal sections 4 weeks after glaucoma induction. Results: Retrograde-Fluorogold-labeled RGC loss caused by persistent glaucoma was decreased by AA. Additionally, AA reduced the postsynaptic current produced by N-methyl-D-aspartate (NMDA) and diminished miniature glutamatergic excitatory neurotransmission to RGCs. On the other hand, AA increased miniature gamma-aminobutyric acid (GABA)-ergic inhibitory neurotransmission to RGCs and enhanced the GABA-induced postsynaptic current. The excitability of the RGC itself was also decreased by AA. RGCs in glaucomatous slices were less excitable because AA decreased their spontaneous action potential frequency and membrane potential, which led to a hyperpolarized condition. Conclusions: AA directly protected RGCs in a chronic glaucoma rat model by lowering their hyperexcitability. To enhance RGCs’ survival and function in glaucoma, AA may be a viable therapeutic drug.

## 1. Introduction

It is estimated that 57.5 million individuals worldwide are affected by primary open-angle glaucoma (POAG), and this number will reach 111.8 million by 2040 [1]. Retinal ganglion cell (RGC) apoptosis is a hallmark of glaucoma, which has a multifactorial cause, and the mechanisms causing glaucomatous neurodegeneration are not fully understood [2,3,4]. Pathologically high intraocular pressure (IOP) is an important but not the only risk factor for glaucoma [2]. Clinical observation has revealed that some individuals still experience visual impairment even after their IOP has been brought under control with medication or surgery, and some people experience normal tension glaucoma [5]. Vision loss in glaucoma is now believed to be based on apoptosis or degenerative changes in RGCs. Therefore, identifying a protective strategy for the RGCs’ somata to inhibit or delay the apoptosis cascade reaction is particularly important.

Asiatic acid (AA), extracted from herbaceous plants, has been shown to possess neuroprotective properties in in vitro and in vivo studies [6,7]. The neuroprotective effect of AA by reducing neurotoxicity, protecting against H_2_O_2_ and reducing mitochondrial dysfunction was further confirmed in experiments using C_2_-ceramide-induced cortical neuron injury in rats, the SH-SY5Y cell line and rotenone-induced cellular damage [8,9,10]. AA may be helpful for the treatment of chronic glaucoma due to its various pharmacological properties. Moreover, the structural characteristics of AA and the oral administration of AA in rodents were not found to have significant toxic effects. Therefore, we aimed to verify whether AA has a neuroprotective effect on RGCs of chronic glaucoma rats.

A key pathogenic component for the onset of glaucoma is excitatory toxic damage. It is crucial to maintain the dynamic equilibrium between inhibitory gamma-aminobutyric acid (GABA) and excitatory glutamate. In the hippocampus CA1 and CA3 region of mice, AA has been found to ameliorate glutamate-induced memory impairment and lessen pyramidal neuron loss [8]. Cortical neurons are shielded from glutamate-induced excitotoxicity by AA and its derivatives [11]. Previous reports have found that AA plays a sedative role by regulating the central GABAergic system [12]. GABA is the most significant inhibitory neurotransmitter in the central nervous system [13,14]. In Xenopus laevis oocytes, AA inhibits the postsynaptic currents induced by GABAA receptors [15]. Meanwhile, few studies investigated the role of AA in regulating the GABAergic system in RGCs.

In this study, the number of surviving RGCs was counted using the retrograde fluorescence labeling method, and the activity and function of the synaptic circuits were examined using the whole-cell patch clamp method. We examined the mechanism of AA’s synaptic modulation and confirmed whether it has a neuroprotective impact on chronic glaucoma RGCs.

## 2. Materials and Methods

### 2.1. Ethical Approval

The Association for Research in Vision and Ophthalmology (ARVO) Statement for the Use of Animals in Ophthalmic and Vision Research was followed during the animal procedures. The experiments were approved by the Ethics Committee for Animal Studies at the Eye and ENT Hospital of Fudan University. The study’s researchers went out of their way to utilize fewer animals and lessen the suffering they endured.

### 2.2. Animals

For all trials, 200 adult male Wistar rats weighing 200–250 g and older than 2 months were used (SLAC Laboratory Animal Co., Ltd., Shanghai, China). At 23 ± 2 °C and 60–70% humidity, the rats were housed in a 12 h light/dark cycle. All rats were anesthetized via the intraperitoneal injection of ketamine and methyl thiazide.

### 2.3. Rat Model of Ocular Hypertension

As previously reported [16,17,18,19], the right eye’s episcleral veins were precisely isolated and cauterized, whereas the left eye received a sham procedure in which the veins were isolated without being cauterized. To prevent postsurgical infection, tobramycin (Tobres; Alcon-Couvreur) (0.3%) was applied. A chronic glaucoma model induced via episcleral veins’ cauterization was shown to have many features, including IOP increase, progressive RGC degeneration, a reduction in aqueous humor outflow, trabecular meshwork damage and alternation in cytokines’ expression, which were observed in glaucomatous patients [20,21].

### 2.4. Fluorogold Retrograde Labeled RGCs and Whole-Mount Retinas

**Figure 1:**
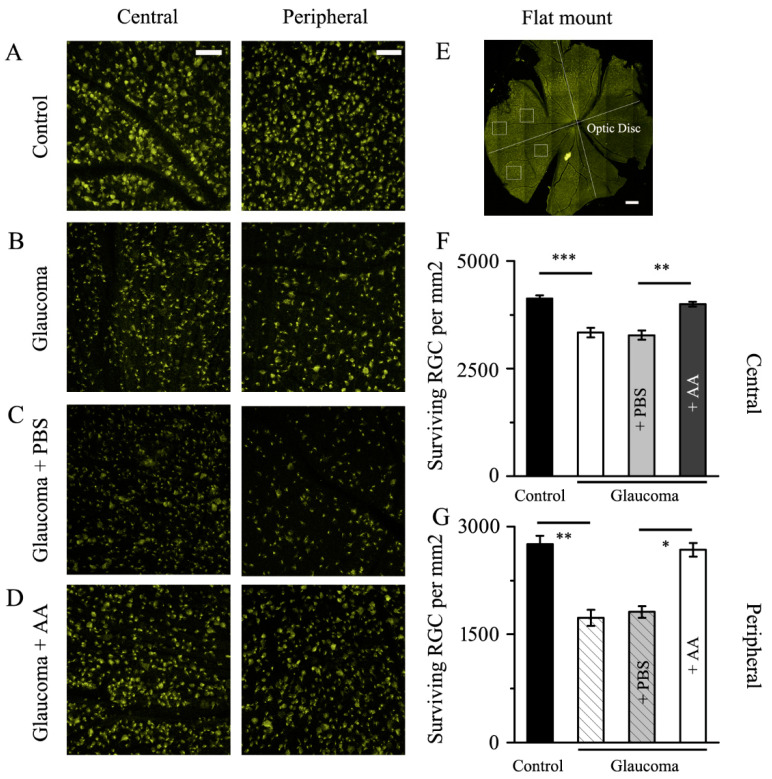
**Fluorogold labeling of RGCs in healthy and glaucomatous retinas with and without AA intervention.** (**A**–**D**) Four weeks after the induction of the glaucoma model, Fluorogold labeling of surviving RGCs in flat-mounted retinas. The central (left) and peripheral (right) panels display enlarged retinal images (scale bar, 50 μm). The control group’s RGC bodies were bright and plump. When compared to PBS (**C**), AA retained cellular integrity and enhanced RGC viability (**D**). (**E**) Whole-mount retina displaying the central and peripheral RGC quantification zones; scale bar, 1 mm. (**F**,**G**) Analysis of RGC survival in glaucomatous eyes with or without PBS or AA treatment and control eyes using quantitative methods. * *p* < 0.05, ** *p* < 0.01, and *** *p* < 0.001 (one-way analysis of variance).

RGCs were retrogradely labeled using the double upper colliculus method. After anesthesia, the rats were fixed to a stereoscope, and the skin was disinfected with 0.5% iodide and cut in the middle to expose the skull. A hole 6 mm posterior and 2 mm lateral to the anterior fontanel was made using a dental drill. A microsyringe was then used to inject 2 μL of 3% Fluorogold into each location, and the needle was left in situ for 5 min. The eyeballs were quickly removed under heavy anesthetic on the 7th day after staining and fixed in 4% paraformaldehyde for an hour. The cornea and lens were removed, the eyeball was cut open 2 mm below the scleral edge of the horn, and the retina was separated and cut into the shape of a four-leaf clover. As seen in Figure 1E, the retina was cut into four equal quadrants, and the ganglion cell layer was pressed against a cover glass slide. The number of RGCs in two locations (0.64 mm × 0.64 mm each) that were 1 and 3 mm from the optic disc were counted blindly in each quadrant. Thus, using laser scanning confocal microscopy (TCS SP8; Leica Microsystems, Germany) with a broadband ultraviolet excitation filter at 20× magnification, the number of RGCs was counted in 16 micrographs per retina.

### 2.5. Preparation of Retinal Slice and Recordings of Electrophysiological

The rat eyeballs were quickly removed and immersed for 30 min in an oxygenated cutting solution that was 95% O_2_/5% CO_2_. The ingredients in this solution were 124 mM sucrose, 3 mM KCl, 1.25 mM NaH_2_PO_4_, 26 mM NaHCO_3_, 3 mM sodium pyruvate, 0.2 mM CaCl_2_, 3.8 mM MgCl_2_ and 10 mM glucose (pH 7.4). The RGC layer was then positioned close to the filter paper by flattening the retinas out on it. Using a manual slicer, the retina was then divided into 200 μm thick slices, which were then incubated in artificial cerebrospinal fluid (ACSF (in mM): 125 NaCl, 3 KCl, 1.25 NaH_2_PO_4_, 26 NaHCO_3_, 2 CaCl_2_, 1 MgCl_2_ and 15 D-glucose (pH 7.35–7.45)) for around 40 min.

We chose RGCs for whole-cell patch clamp based on their location, morphology and Lucifer yellow staining using an IR-DIC-sensitive charge-coupled device (CCD) camera system (Nikon, Tokyo, Japan) and a water-immersion objective lens at 40× magnification [22]. At −70 mV, the holding potential was set. The recordings were made using a personal computer running Clampex and Clampfit software (version 10, Axon Instruments), an Axopatch MultiClamp 700B amplifier (Axon Instruments, Foster City, CA, USA; sampling frequency = 10 kHz, filter frequency = 1 kHz) and a Digidata 1440A digital analog converter system (Axon Instruments, Foster City, CA, USA).

Excitatory postsynaptic currents (EPSCs) were captured using 3–5-MΩ electrodes filled with a solution consisting of (in mM) 120 CsMeSO_3_, 5 NaCl, 0.2 GTP-Na, 2 ATP-Mg, 10 TEA-Cl, 2 EGTA and 10 HEPES (pH 7.2; adjusted with CsOH) in the whole-cell configuration in voltage clamp mode.

N-methyl-D-aspartate (NMDA) was puffed for 8 s using a small-tipped (2 μm) pipette that was brought to within 50 μm of the RGC soma being recorded using a pressure feeding device (Picospritzer, General Valve Corp., Fairfield, NJ, USA) as previously described [23].

The fluid in the electrodes used to record mEPSCs and NMDA-induced postsynaptic currents had the same chemical make-up. The recorded extracellular fluid was replaced with ACSF containing the following (in mM): 125 NaCl, 3 KCl, 1.25 NaH_2_PO_4_, 15 D-glucose, 2 CaCl_2_, 26 NaHCO_3_ and 0.02 ascorbic acid (pH 7.35–7.45), because magnesium ions inhibit the NMDA receptor. 6-cyano-7-nitroquinoxaline-2,3-dione (CNQX; 10 μM), 2-(3-carboxypropyl)-3-amino-6-methoxyphenyl-pyridazinium bromide (SR95531; 10 μM), strychnine (5 μM) and tetrodotoxin (TTX, 1 μM) were used during the recording process.

An Axopatch MultiClamp 700B amplifier was used to clamp the holding potential at −70 mV in order to record inhibitory postsynaptic currents (IPSCs). When filled with an intracellular solution containing (in mM) 150 CsCl, 10 HEPES, 1 MgCl_2_, 1 EGTA, 0.1 CaCl_2_, 4 Mg-ATP and 0.4 Na-GTP (pH 7.2), the patch pipettes had open-tip resistances of 4–8 MΩ. To prevent fast Na+ currents, ligdocaine N-ethyl bromide (QX314; 2.0 mM) was added to the pipette solution.

As previously stated [23], GABA was puffed for 8 s through a small-tipped (2 μm) pipette using a pressure feeding system (Picospritzer, General Valve Corp., Fairfield, NJ, USA) that was moved under visual control to within 50 μm of the RGC soma being recorded. The fluid in the electrodes used to record GABA-induced postsynaptic currents had the same chemical make-up (mIPSCs). Strychnine (5 μM), D-2-amino-5-phosphonovalerate (D-AP5; 10 μM), CNQX (10 μM) and TTX (1 μM) were added to the bath during the recording process.

A solution containing (in mM) 120 potassium D-gluconate, 10 phosphocreatine, 0.3 GTP-Na, 4 ATP-Mg, 1 EGTA, 10 HEPES, 1 MgCl_2_ and 0.1 CaCl_2_ was used to record action potentials in the whole-cell configuration (pH 7.2; adjusted with KOH).

### 2.6. Drug Administration

For four weeks straight, the tip of a needle was injected once a week into the superior hemisphere of the eye at a 45° angle through the sclera and into the vitreous body. A 2 μL injection of 10 μM AA was given intravitreally to some rats. Then, 2 μL of PBS was injected intravitreally into the control eyes, and this procedure was repeated every week after that. For patch clamp recordings, the following medications were used: 10 μM AA, 1 μM TTX (to abolish spontaneous action potentials), 10 μM CNQX and 50 µM D-AP5 (to block ionotropic glutamate receptors), 10 μM SR95531 (to block GABA_A_ receptors) and 5 µM strychnine (to block glycine receptors). Sigma-Aldrich was used for all drug purchases.

### 2.7. Data Analysis

The mean and standard error of the mean (SEM) were used to express data. A two-sample *t*-test was used to statistically compare the mean frequency and amplitude before and after medication delivery. A one-way ANOVA with Bonferroni post hoc tests for multiple comparisons was used to evaluate the data provided. SPSS software was used to conduct the analysis (17.0; SPSS Inc., Chicago, IL, USA). A significant difference was shown by the symbol * *p* < 0.05.

## 3. Results

### 3.1. AA Reduced the RGCs Loss Induced by Chronic Glaucoma

In line with our prior findings, the 4-week chronic glaucoma group’s much lower RGC count than the control group was brought on by high IOP [16,24]. At 0, 1, 2 and 3 weeks after glaucoma modeling, AA intravitreal injections were carried out. The Fluorogold labeling experiment was performed in the 3rd week after glaucoma modeling, and RGC counts images were performed in the 4th week after glaucoma modeling. RGC density was measured in two locations in each quadrant, as seen in Figure 1E. Compared with the control group, central and peripheral RGCs decreased significantly in the glaucoma group and glaucoma + PBS group (all *p* < 0.01, Figure 1A,B,F,G). In comparison to the glaucoma group and the glaucoma + PBS group, the number of RGCs that survived in the glaucoma + AA intervention group was significantly higher in both the central and peripheral regions (all *p* < 0.05, Figure 1B–D,F,G).

### 3.2. AA Decreased the Frequency and Amplitude of mEPSCs in Glaucomatous RGCs

**Figure 2:**
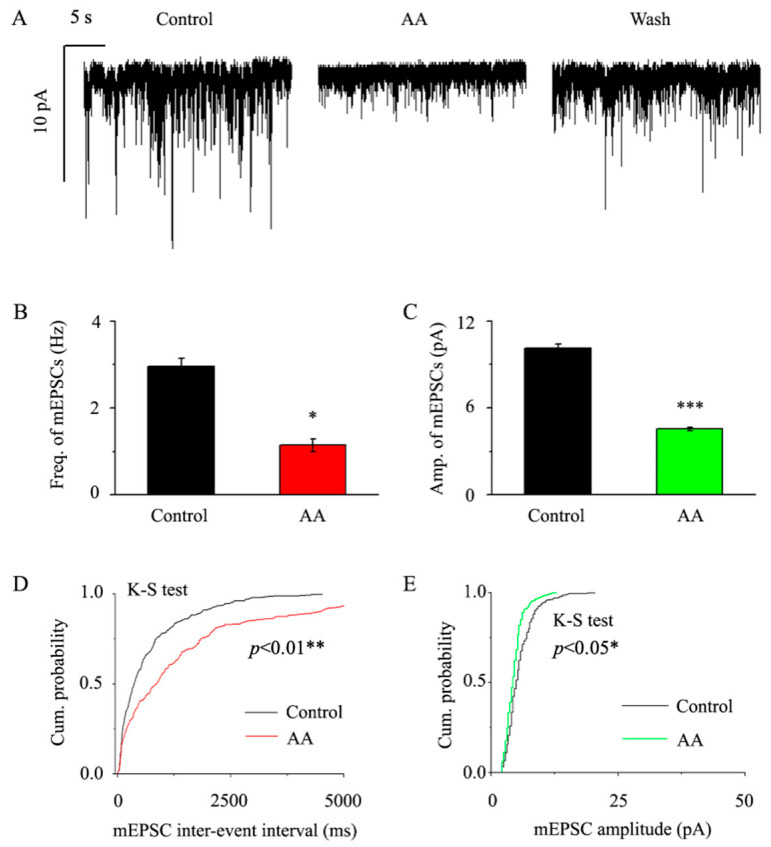
**AA decreased the frequency and amplitude of glutamatergic mEPSCs in glaucomatous RGCs.** (**A**) Representative large-scale traces depicting the effect of 10 μM AA on mEPSCs during AA treatment (middle), recovery (right) and control conditions (left). Horizontal scale bar, 5 s; vertical scale bar, 10 pA. (**B**,**C**) Summarized data (n = 14; from ten slices) on the frequency (**B**) and amplitude (**C**) of glutamatergic mEPSCs. (**D**,**E**) A typical neuron’s cumulative frequency and amplitude distributions of glutamatergic mEPSCs under control and during AA treatment demonstrate that AA dramatically altered the cumulative frequency curve to the right and the cumulative amplitude curve to the left. Student’s paired t test; * *p* < 0.05, ** *p* < 0.01 and *** *p* < 0.001 in comparison to the control conditions.

We preincubated the retinal slices with 1 μM TTX + 10 μM SR95531 + 10 μM strychnine in order to examine the vesicular quantum release of glutamatergic synaptic transmitters, and then, we perfused AA to reduce the frequency and amplitude of mEPSCs in RGCs (Figure 2A). Glaucoma retinal RGCs had a basic mEPSC frequency of 2.95 ± 0.2 Hz, whereas AA caused that frequency to drop to 1.14 ± 0.15 Hz (*p* < 0.05, n = 14, Figure 2B). Additionally, AA reduced the mEPSC amplitude from 10.12 ± 0.32 pA to 4.55 ± 0.12 pA (*p* < 0.001, n = 14, Figure 2C). We examined and compared the effects of AA on the cumulative frequency and cumulative amplitude of glutamatergic mEPSCs using the Kolmogorov–Smirnov (K-S) test. AA significantly shifted the cumulative frequency curve to the right (n = 11, *p* < 0.01, Figure 2D) and the cumulative amplitude curve to the left (n = 11, *p* < 0.05, Figure 2E). AA took approximately 4 min to take effect, reached maximum effect in 8 min, and lasted for 20 min before full recovery. After an ACSF wash, the synaptic current was returned to the control level at the end of the experiment.

### 3.3. AA Reduced the Synaptic NMDA-Induced Current Amplitude

**Figure 3:**
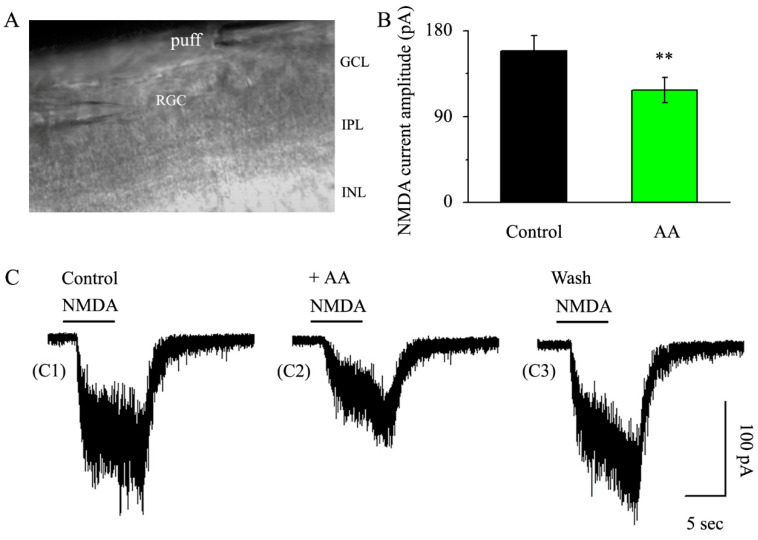
**AA reduced the NMDA-induced current amplitude in glaucomatous RGCs.** (**A**) Infrared interferometric phase contrast microscopy showed three layers of the inner retina, the inner nuclear layer, the inner plexiform layer and the ganglion cell layer. The recording electrode of the encapsulated cell on the left side and the puff delivery electrode on the right side. (**B**) NMDA-induced postsynaptic receptor current amplitude in control conditions and after AA administration. (**C**) Representative large-scale traces demonstrating the effect of 10 μM AA on NMDA-induced postsynaptic receptor current under control conditions (C1), AA treatment (C2) and recovery conditions (C3). Scale bars: vertical, 100 pA; horizontal, 5 s. Student’s paired t test; ** *p* < 0.01 in comparison to the control conditions. GCL, ganglion cell layer; IPL, inner plexiform layer; INL, inner nuclear layer.

The ability of AA to decrease the frequency and amplitude of mEPSCs suggested that postsynaptic receptors might be involved in this regulation. Therefore, we examined whether AA regulates postsynaptic NMDA receptor currents. The NMDA receptor current was systematically induced by an 8 s pressure injection every 3 min at a distance of 50 μm from the recorded RGC (Figure 3A). In the control condition, the amplitude of the NMDA current was 158.8 ± 16.21 pA. After AA was applied with continuous gravity irrigation for 5 min, the amplitude of the NMDA current decreased to 117.9 ± 13.5 pA (*p* < 0.01, n = 11, Figure 3B,C), and the NMDA receptor current recovered completely after the washout of AA.

### 3.4. AA Enhanced the Frequency and Amplitude of GABAergic mIPSCs in Glaucomatous RGCs

**Figure 4:**
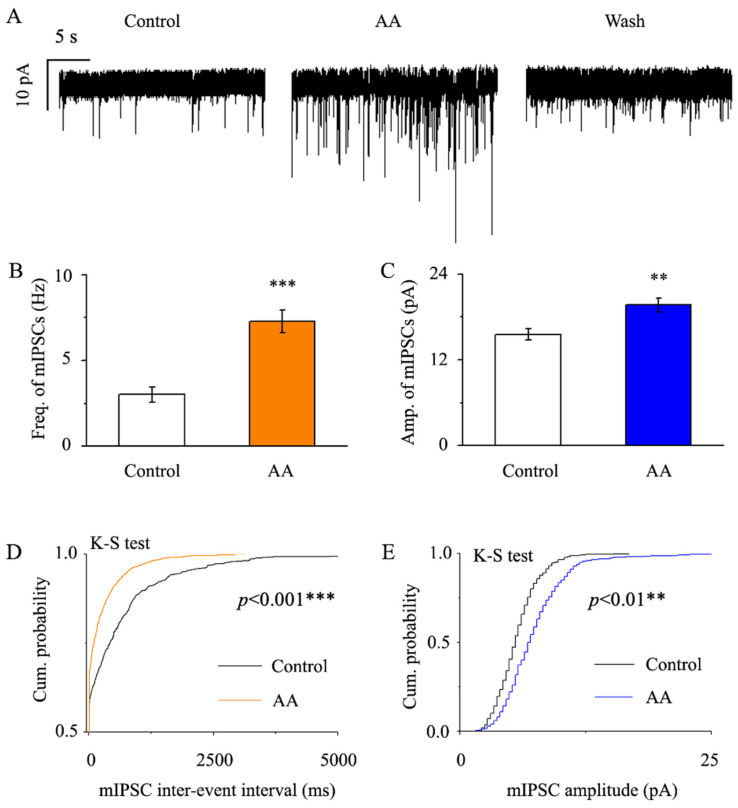
**AA decreased the frequency and amplitude of GABAergic mIPSCs in glaucomatous RGCs.** (**A**) Representative large-scale traces depicting the effect of 10 μM AA on mIPSCs during AA treatment (middle), recovery (right), and control conditions (left). Scale bars: vertical, 10 pA; horizontal, 5 s. (**B**,**C**) Summarized data (n = 15; from nine slices) on the frequency (**B**) and amplitude (**C**) of GABAergic mIPSCs. (**D**,**E**) Under control conditions and during AA treatment, the cumulative frequency and amplitude distributions of GABAergic mIPSCs in a sample neuron showed that AA considerably shifted the cumulative frequency curve to the left and the cumulative amplitude curve to the right. Student’s paired t test; ** *p* < 0.01 and *** *p* < 0.001 in comparison to the control conditions.

Amounts of 1 μM TTX + 10 μM CNQX + 50 μM AP-5 + 10 μM strychnine were preincubated with RGCs before being perfused with AA, and AA enhanced the frequency and amplitude of mIPSCs in RGCs (Figure 4A). Glaucoma RGCs had a baseline mIPSC frequency of 3.01 ± 0.45 Hz; however, AA increased the frequency to 7.28 ± 0.67 Hz (*p* < 0.001, n = 15, Figure 4B), as well as the amplitude, which was increased from 15.55 ± 0.81 pA to 19.66 ± 0.97 pA (*p* < 0.01, n = 15, Figure 4C). We analyzed and compared the effects of AA on the cumulative frequency and cumulative amplitude of glutamatergic mEPSCs using the Kolmogorov–Smirnov (K-S) test. AA significantly shifted the cumulative frequency curve to the left (n = 13, *p* < 0.001, Figure 4D) and the cumulative amplitude curve to the right (n = 13, *p* < 0.01, Figure 4E). These effects took place within 4 min, reached a maximum in 8 min and lasted for 20 min before full recovery. After an ACSF wash, the synaptic current was returned to the control level at the end of the experiment.

### 3.5. AA Enhanced the Synaptic GABA-Induced Current Amplitude

**Figure 5:**
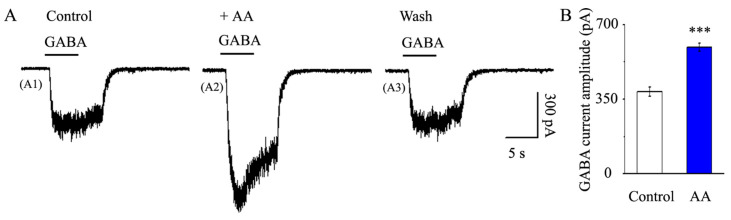
**AA increased the GABA-induced current amplitude in glaucomatous RGCs.** (**A**) Representative large-scale traces demonstrating the effect of 10 μM AA on GABA-induced postsynaptic receptor current during AA treatment (**A2**), recovery (**A3**) and control conditions (**A1**). Scale bars: vertical, 300 pA; horizontal, 5 s. (**B**) The amplitude of the postsynaptic receptor current generated by GABA under control and after AA treatment. Student’s paired *t*-test; *** *p* < 0.001 compared with the control conditions.

We then examined GABA postsynaptic receptor function. We chose 30 μM as the GABA concentration employed in this work based on its EC_50_ value and the concentration-dependent results of GABA-induced postsynaptic currents in our earlier articles. An 8 s pressure injection every 3 min at a distance of 50 μm from the recorded RGC was used to methodically produce the GABA receptor current (Figure 3A). The GABA current was 385.9 ± 21.22 pA in the control condition, and after applying AA via continuous gravity irrigation for 5 min, the GABA current was 592.65 ± 19.11 pA (*p* < 0.001, n = 9, Figure 5A,B). After AA washout, the GABA receptor current fully returned.

### 3.6. AA Reduced the Spontaneous Action Potential Frequency in RGCs

**Figure 6:**
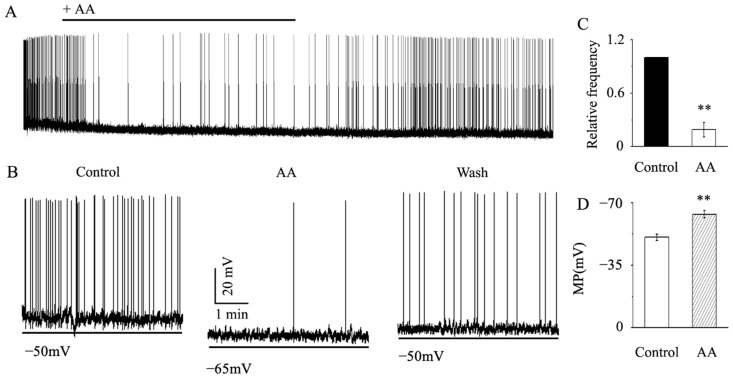
**AA decreased glaucomatous RGC action potential frequency.** (**A**) Representative recordings showing that AA decreased glaucomatous RGC action potential frequency. (**B**) Typical large-scale traces demonstrating how 10 μM AA affected the action potential and membrane potential of RGCs under control conditions (left), AA treatment (center) and recovery conditions (right). Horizontal scale bar, 1 min; vertical scale bar, 20 mV. (**C**) The frequency of action potentials under control conditions and following AA administration. (**D**) Membrane potential under control conditions and following AA administration. Student’s paired t test; ** *p* < 0.01 in comparison to the control conditions.

AA decreases the activity of the excitatory glutamatergic system and enhances the function of the inhibitory GABAergic system. Therefore, is the total effect of AA on postsynaptic RGCs excitatory or inhibitory? To answer this question, we further examined the effect of AA on the spontaneous action potentials of RGCs. As shown in Figure 6, AA reduced the RGC action potential frequency to 19% ± 8% of the control level (*p* < 0.01, n = 9, Figure 6A–C). AA also reduced the membrane potential of RGCs from −50.6 ± 1.7 mV to −63.5 ± 2.1 mV after AA treatment (*p* < 0.01, n = 12, Figure 6A,B,D). These effects were reversible, and the cells returned to a normal state after washout.

## 4. Discussion

The main purpose of this paper was to demonstrate the synaptic regulatory mechanism by which AA increases RGC survival in chronic glaucoma. We first found that AA decreased presynaptic miniature glutamatergic (excitatory) neurotransmission and increased presynaptic miniature GABAergic (inhibitory) neurotransmission to RGCs. Second, AA decreased postsynaptic NMDA-induced whole-cell currents and increased postsynaptic GABA-induced whole-cell currents. Finally, AA decreased the frequency at which action potentials fired, reducing the excitability of RGCs in glaucomatous retinal slices.

We found that the intravitreal injection of AA in hypertensive rat eyes, which were induced via episcleral veins’ cauterization, may reduce apoptosis or degenerative changes in RGCs. In an ocular hypertension model created by injecting magnetic beads into the anterior chamber, our research team previously discovered that low concentrations of AA were unable to significantly increase the number of RGCs, whereas both 10 μM and 100 μM greatly enhanced the number of surviving RGCs [25]. Therefore, we settled on a medication concentration of 10 μM. Fluorogold retrograde labeling is a very effective and feasible method for monitoring the number of surviving RGCs in basic animal experiments [26]. First, for the effective retrograde labeling of RGCs, robust axoplasmic flow is necessary [27]; therefore, damage to any part of this pathway directly affects the number of RGC bodies labeled with Fluorogold [26,28]. Second, retrograde labeling is also sensitive to RGC loss and functional impairment. When RGCs are lost or damaged under pathological conditions, the cell bodies and axons of RGCs are affected accordingly, preventing retrograde dyes from labeling these damaged RGCs. Glaucoma is a complex illness, as is well known. On the one hand, our study concentrated on the harm that excitotoxicity does to RGCs. On the other hand, axonal transport and neurotrophic factor supply are also important factors [29]. Therefore, a reduction in the number of tagged RGCs may have been caused by reduced axonal transport. One of the main characteristics of glaucoma is RGC axonal degeneration. The partial loss of retinal nerve fibers in patients with glaucoma suggests that axonal degeneration may aggravate RGC death [30]. Therefore, apart from neutralizing RGC excitotoxic damage by enhancing the activity of the inhibitory neurotransmitter GABAergic system, AA may improve the viability of RGCs by improving the efficiency of axonal transport and providing more nutrient support. Although damaged RGCs will eventually die, AA regulates axonal transport’s capacity to slow down the death process. Therefore, whether AA protects RGCs by improving the axonal transport capacity will be further explored in our follow-up experiments.

According to previous reports, glutamate-mediated overactivation contributes to some of the damage caused by glaucoma. This overactivation further activates downstream catabolic enzymes after an increase in intracellular free calcium, which starts cytotoxic cascade reactions. For instance, pretreatment with AA (0.1–100 nmol/L) reduced the cytotoxicity brought on by 10 mmol/L glutamate in SH-SY5Y cell lines. It was discovered that AA’s effects on mitochondria and oxidative stress contributed to its partial protective mechanism. As a result of dramatically lowering reactive oxygen species (ROS) and stabilizing the mitochondrial membrane potential (MMP), AA prevented cells from going into apoptosis [8]. This protective effect is consistent with our results. We further elucidated the regulatory mechanism of AA from the perspective of synaptic circuits: AA decreased the magnitude of current produced by NMDA receptors in the postsynaptic membrane and hindered the release of glutamate, an excitatory neurotransmitter.

We discovered that AA increased the frequency of GABAergic mIPSCs while decreasing the frequency and amplitude of glutamatergic mEPSCs. This increase in frequency is indicative of an increase in the numbers of neurotransmitters released presynaptically. On one hand, the increase in amplitude may be due to the cumulative effect of the increase in presynaptic release frequency, leading to the summation of the currents; on the other hand, an increase in amplitude can also suggest that the regulations are directly regulated by postsynaptic receptors [23]. AA has a regulatory effect on the excitatory synaptic transmission of Schaffer commissural-CA1 synapses, which is caused by direct or indirect effects of AA on GABAergic receptors. AA acts specifically on GABAB receptors but not GABAA receptors in the rat hippocampus, increasing potassium conductance and causing potassium ions to flow out of neurons, leading to the hyperpolarization of neurons and reducing voltage-dependent calcium conductance, thereby reducing the excitatory postsynaptic potential (EPSP) [31,32]. Another study on hippocampal learning and memory reached a conclusion inconsistent with ours, finding that AA significantly increased spontaneous EPSC amplitude mainly through the AMPA glutamate receptor but had no effect on the GABAergic system [33]. However, in our experimental conditions, we demonstrated that AA enhanced the activity of the GABAA system in the retina of chronic glaucoma rats, including enhancing the release of GABA from presynaptic inhibitory neurons and enhancing the current induced by GABAA-type receptors distributed on the postsynaptic membrane. Furthermore, AA decreased the magnitude of current produced by NMDA receptors in the postsynaptic membrane and prevented the release of glutamate, an excitatory neurotransmitter. In this antagonistic transmitter system pair, AA acts dynamically via affecting both the excitatory and inhibitory systems to maintain the dynamic balance of the neurons.

Because AA differentially regulates the two major neurotransmitter systems projecting to RGCs, we examined the ultimate effect on RGCs. Field EPSPs (fEPSPs) induced by the electrical stimulation of Schaffer commissural fibers were recorded in the CA1 region of the central hippocampus, where a similar mechanism has been reported. AA had a dose-dependent inhibitory effect on fEPSPs, with an IC50 (half maximal inhibitory concentration) of 14 μM, which is comparable to the effective concentration of the drug in our experiment [31]. By directly recording RGC action potentials, we found that AA significantly reduced the action potential frequency.

Our results show that AA might be useful in prolonging the survival of RGCs by lowering hyperexcitability at the synaptic circuit level. However, whether AA could rescue the apoptosis of RGCs needs further investigations.

## 5. Conclusions

By lowering RGCs’ hyperexcitability at the synaptic circuit level in a chronic glaucoma rat model, AA directly protected RGCs. For enhancing RGC survival and function in glaucomatous neurodegeneration, AA may be a viable therapeutic drug.

## Data Availability

The data presented in this study are available in the manuscript.
